# Editorial: Insights in neurogenomics: 2022

**DOI:** 10.3389/fgene.2023.1252582

**Published:** 2023-08-07

**Authors:** Aayushi Gandhi, Sarah H. Elsea

**Affiliations:** Department of Molecular and Human Genetics, Baylor College of Medicine, Houston, TX, United States

**Keywords:** rare disease, genetic diagnosis, therapeutic intervention, early diagnosis, genetic testing

Rare diseases are often thought of as clinically complex, hard to diagnose, difficult to treat, and poorly characterized in healthcare settings worldwide. Although rare diseases may not individually affect large numbers of people, there are over 10,000 rare disorders that collectively affect over 300 million people worldwide, which has resulted in the official recognition of rare disease as a public health issue (Spelbrink et al.). Genetic disorders have also become one of the leading causes of infant mortality and general morbidity in wealthy industrialized countries (Wojcik et al., 2019). Consequently, interest in human genetics is rapidly increasing, and early diagnosis of rare genetic diseases has become a major topic of interest (Wojcik et al., 2019). The early diagnosis and detection of rare genetic disorders is critical because it leads to both earlier intervention and earlier treatment, which are key to improving patient outcomes. Currently, the most prominent strategy for early diagnosis of rare genetic disease is newborn screening. Avoiding harmful environmental exposures that may increase mutation frequency is another strategy to reduce disease risk, as well as lifestyle changes, even although lifestyle changes for disease prevention are often difficult across populations (Mort et al., 2008; Nielsen et al., 2017). That said, recent studies have demonstrated that these approaches are inadequate in their attempts to identify and diagnose genetic disorders early enough to significantly reduce disease and implement early treatment strategies (Wojcik et al., 2019). The Research Topic, *Insights in neurogenomics,* brings together studies of genetic neurological conditions that collectively highlight the need for early, efficient, and accurate diagnosis.

Gan et al. investigated two rare diseases, Duchenne muscular dystrophy (DMD, MIM #310200) and Becker muscular dystrophy (BMD, MIM #300376), hereditary neuromuscular recessive disorders caused by pathogenic variants in the dystrophin gene. At Hunan Children’s Hospital, 150 male patients diagnosed with either BMD or DMD were studied to understand the effect of dystrophin gene variants on patient outcomes. Data were collected for an average of 3.42 years, and the severity of disease progression was measured by tracking patients’ loss of ambulation (LOA). According to the Cox regression analysis, risk factors for early LOA in patients with DMD included positive family history, short duration of glucocorticoid (GC) treatment, and frameshift mutation. The mean age of LOA for the 150 patients studied was 10.4 years, and 60.7% of these patients ended up receiving glucocorticoid (GC) treatment at a mean age of 7.0 ± 2.7 years. Individuals with DMD who received GC treatment after 7 years of age were at higher risk for early LOA than those who received treatment earlier. Delay time from symptom onset to diagnosis and delay time from diagnosis to receiving GC treatment were carefully studied to determine the cause of variation in LOA among patients. Cox multivariate analysis suggested that the time of initiation of GC therapy did, in fact, play a significant role delaying LOA. With regards to treatment strategies, data suggested that a vast majority of the patients (>90%) with single nucleotide or short indel variants were predicted to benefit from exon-skipping therapies, which indicates that early molecular diagnosis could have led to more timely personalized treatment strategies being implemented (Gan et al.).

A study by Yongsheng et al. drew similar conclusion in studying amyloid transthyretin (ATTR) amyloidosis (MIM #105210). ATTR amyloidosis is the pathologic accumulation of extracellular protein due to misfolding and aggregation of unstable transthyretin monomers which form insoluble fibrils resistant to proteolysis. ATTRv is a very rare autosomal dominant disease which primarily presents in the form of cardiac or neuropathic disorders and often becomes fatal within 7–12 years of symptom onset. Currently, there are only rough estimates of the prevalence of ATTRv in China. In this study, analysis of *TTR* variants from the Genome Aggregation Database (gnomAD) and two genomic sequencing databases (ChinaMAP and Amcarelab) suggested that the prevalence of ATTRv in China is significantly underestimated. Using variant data extracted from these databases, Yongsheng et al. were able to calculate pathogenic variant allele frequency and estimate the prevalence of ATTRv in the pan-ethnic populations and specifically in the mainland Chinese population. The prevalence of ATTRv worldwide was estimated to be about 57.4/100,000, and the prevalence of ATTRv in China was estimated to be between 18.9 and 74.9/100,000. The authors concluded that the prevalence in China is far higher than previous estimates but similar to that of other populations. The high and early fatality associated with ATTRv can pose a heavy financial burden for affected families and society. Thus, the failure of traditional clinical diagnostic methods to produce reliable estimates of ATTRv in the past highlights the importance of developing a more effective method for early diagnosis (Yongsheng et al.).

One possible avenue for research into early diagnostic methods includes the utility of biomarkers. A study by Liang et al. reviewed investigations into both brain age and biological age and approaches to capture these parameters of aging. The unexpected acceleration of both brain and biological age is associated with the development of Alzheimer’s disease (AD), and a variety of biomarkers associated with both brain age and biological age can provide insight on an individual’s health and potential trajectory towards developing AD. When an individual’s brain age is elevated, they may experience a noticeable decrease in cognitive functioning. Brain age gaps have also been used to differentiate between progressive and stable mild cognitive impairment (MCI) and to predict the progression from MCI to Alzheimer’s disease. Determining biological age involves the analysis of DNA methylation changes to determine an individual’s epigenetic clock and predicted epigenetic age. These clocks help determine changes in the rate of biological aging, which then helps identify the real impact of active aging interventions on patients. Currently, approximately 5 million Americans live with MCI and 6.5 million Americans with AD (Liang et al.). This number is predicted to reach 13.8 million by 2060. Thus, it is critical for early treatment and diagnosis strategies, such as measures of brain and biological age, to be developed quickly. Although measures of brain and biological age have yet to be widely adopted into clinical trials, they may be promising biomarkers for future analysis of both MCI and AD. More research is needed to determine if this divergence is both clinically actionable and meaningful and whether improved brain and biological age results in positive brain health changes (Liang et al.).

Though there is a critical demand for genetic-based diagnostic methods for rare diseases, strategies for how to approach and rectify this are still being developed. One effective approach to collecting global data for rare disease research is illustrated by a study conducted by Spelbrink et al. Patient medical records collected by Ciitizen with help from the TESS Research Foundation were assessed to characterize the neurologic and clinical laboratory phenotype of SLC13A5 citrate transporter disorder (MIM #615905, developmental and epileptic encephalopathy 25 with amelogenesis imperfecta; DEE25), a rare, autosomal recessive genetic disorder. This study analyzed the genotype, clinical phenotypes, and laboratory data of 15 patients from these records and provided valuable insight into ways future studies can ensure that data collection occurs in a comprehensive and timely manner that encourages the implementation of early treatment strategies. All 15 patients studied exhibited both global developmental delay and epilepsy. The majority also experienced difficulties in communication. While many exhibited movement disorders such as ataxia and dystonia, many individuals did still attain their motor milestones continuously, though much later than most of their peers. All 15 patients had no documented renal, liver, or hematologic abnormalities. Serum citrate levels were measured in only 3 patients, all of which were noticeably elevated. Brain MRIs were documented for 14 out of 15 patients, 50% of which had at least one normal brain MRI. Ultimately, the results of the analysis of these 15 patients indicated that both the epilepsy phenotype and SLC13A5 citrate transporter disorder impacted global development. Most noticeably, this came in the form of motor, coordination, and communication difficulties. Due to the rarity of the disease and the challenges in characterizing clinical features, the TESS Research Foundation asked families to enroll in the Ciitizen platform to allow their medical records to be uploaded to a cloud-based data platform for data collection, extraction, and analysis. This approach allowed for world-wide collaboration among patient advocacy groups, academic groups, and industry groups, a strategy that will be crucial for development of treatment for this disorder and other rare genetic disorders in the future (Spelbrink et al.).

Overall, these studies serve to emphasize the value that diagnosing rare genetic diseases in their early stages can have on the treatment options available to patients as well as overall patient outcomes. With many different avenues for early diagnosis including biomarkers, it is crucial to establish a framework for recruiting and enrolling individuals with rare diseases worldwide in order to collect enough data for appropriate investigations and eventually solutions. Additionally, it is crucial to direct efforts toward developing more effective screening processes, especially for high-risk individuals. The more that is known about the 10,000+ genetic disease in this world, the easier it will be to reduce the burden of this public health issue and improve the quality of life of at-risk individuals worldwide.

## References

[B1] Mort M. Ivanov D. Cooper D. N. Chuzhanova N. A. (2008). A meta-analysis of nonsense mutations causing human genetic disease. Hum. Mutat. 29 (8), 1037–1047. 10.1002/humu.20763 18454449

[B2] Nielsen J. B. Leppin A. Gyrd-Hansen D. E. Jarbøl D. E. Søndergaard J. Larsen P. V. (2017). Barriers to lifestyle changes for prevention of cardiovascular disease – A survey among 40-60-year-old Danes. BMC Cardiovasc. Disord. 17, 245. 10.1186/s12872-017-0677-0 28899356 PMC5596487

[B3] Wojcik M. H. Schwartz T. S. Thiele K. E. Paterson H. Stadelmaier R. Mullen T. E. (2019). Infant mortality: The contribution of genetic disorders. J. Perinatology 39 (12), 1611–1619. Article 12. 10.1038/s41372-019-0451-5 PMC6879816 31395954

